# Endothelial dysfunction in cardiovascular diseases: mechanisms and in vitro models

**DOI:** 10.1007/s11010-025-05289-w

**Published:** 2025-04-21

**Authors:** Ana Grego, Cristiana Fernandes, Ivo Fonseca, Marina Dias-Neto, Raquel Costa, Adelino Leite-Moreira, Sandra Marisa Oliveira, Fábio Trindade, Rita Nogueira-Ferreira

**Affiliations:** 1 RISE-Health, Department of Surgery and Physiology, Faculty of Medicine, University of Porto, Alameda Prof. Hernâni Monteiro, 4200-319 Porto, Portugal; 2 LAQV-REQUIMTE, Department of Chemistry, University of Aveiro, 3810-193 Aveiro, Portugal; 3 Department of Angiology and Vascular Surgery, Unidade Local de Saúde de São João, Alameda Prof. Hernâni Monteiro, 4200-319 Porto, Portugal; 4 Universidade Católica Portuguesa, CBQF-Centro de Biotecnologia e Química Fina-Laboratório Associado, Escola Superior de Biotecnologia, Rua Diogo Botelho 1327, 4169-005 Porto, Portugal; 5 Department of Cardiothoracic Surgery, Unidade Local de Saúde de São João, Alameda Prof. Hernâni Monteiro, 4200-319 Porto, Portugal

**Keywords:** Endothelial cell dysfunction; in vitro models; Chemical/Mechanical stimulation; Cardiovascular diseases

## Abstract

Endothelial cells (ECs) are arranged side-by-side to create a semi-permeable monolayer, forming the inner lining of every blood vessel (micro and macrocirculation). Serving as the first barrier for circulating molecules and cells, ECs represent the main regulators of vascular homeostasis being able to respond to environmental changes, either physical or chemical signals, by producing several factors that regulate vascular tone and cellular adhesion. Healthy endothelium has anticoagulant properties that prevent the adhesion of leukocytes and platelets to the vessel walls, contributing to resistance to thrombus formation, and regulating inflammation, and vascular smooth muscle cell proliferation. Many risk factors of cardiovascular diseases (CVDs) promote the endothelial expression of chemokines, cytokines, and adhesion molecules. The resultant endothelial activation can lead to endothelial cell dysfunction (ECD). In vitro models of ECD allow the study of cellular and molecular mechanisms of disease and provide a research platform for screening potential therapeutic agents. Even though alternative models are available, such as animal models or ex vivo models, in vitro models offer higher experimental flexibility and reproducibility, making them a valuable tool for the understanding of pathophysiological mechanisms of several diseases, such as CVDs. Therefore, this review aims to synthesize the currently available in vitro models regarding ECD, emphasizing CVDs. This work will focus on 2D cell culture models (endothelial cell lines and primary ECs), 3D cell culture systems (scaffold-free and scaffold-based), and 3D cell culture models (such as organ-on-a-chip). We will dissect the role of external stimuli—chemical and mechanical—in triggering ECD.

## Introduction

Endothelial cells (ECs) are thin and elongated cells [1] forming the inner lining of the vascular wall of every blood vessel, from large arteries—macrocirculation—to small capillaries—microcirculation—and of lymphatic vessels. These cells are essential to maintain blood vessel integrity and blood fluidity [2]. ECs are arranged side-by-side, creating a semi-permeable monolayer, the endothelium [3]. A healthy endothelium acts as a selective barrier to molecules, controlling their exchange between the blood and tissues to ensure homeostasis [4]. Due to their position, ECs are also the first barrier for all circulating cells and pathogens, being exposed to changes in blood composition and flow. ECs are, thus, key players in the vasculature, sensing biomechanical and biochemical factors, which can induce rapid, short-term, and long-term adaptation processes [5–9]. ECs differ morphologically, physiologically, and phenotypically among the different types of blood vessels and according to the organ [10, 11]. There is a high heterogeneity of ECs’ surface and cytoplasmic markers [9]. Both CD31 [12] and VE-cadherin [13] are universal ECs’ markers. Ephrin type-B receptor 2 is a specific marker of arterial ECs [14]. Cultured human umbilical vein ECs (HUVECs), in particular, express typical molecular markers, including CD31, von Willebrand factor (VWF), CD34, intercellular adhesion molecule-1 (ICAM-1), E-selectin, vascular cell adhesion molecule-1 (VCAM-1), and angiotensin I converting enzyme (ACE) [15, 16] (Table 1).

**Table 1:** Markers of endothelial cells [17, 18]

Marker	Endothelial cell type
VE-statin	Embryonic ECs
CD31/PECAM-1	Non-specific ECs
CD144/VE-cadherin	Non-specific ECs
CD146/MCAM	Non-specific ECs
CD54/ICAM-1	ECs marker in sites of inflammation
EphB4	Adult venous ECs
VWF	HUVECs
CD105/Endoglin	Proliferating vascular ECs
CD62P/P-selectin	Vascular ECs
CD102/ICAM-2	Vascular ECs
CD141/Thrombomodulin	Vascular ECs
VEGFR2	Vascular ECs and EPCs
EphrinB2	Arterial ECs
Podoplanin	Lymphatic ECs
LYVE-1	Lymphatic ECs
CD62E/E-selectin	ECs after stimulation by TNF-α and IL-1β
CD44	ECFCs
CD34	EPCs and HUVECs
CD133/Prominin-1	EPCs
TEM8	Tumor vasculature
TNAP	ECs of brain blood vessels

*ECs* endothelial cells, *ECFCs* endothelial colony-forming cells, *EPCs* endothelial progenitor cells, *EphB*4 ephrin type-B receptor 4, *HUVECs* human umbilical vein endothelial cells, *ICAM*−1 intercellular adhesion molecule-1, *ICAM*−2 intercellular adhesion molecule-2, *IL*−1*β* interleukin-1β, *LYVE*−1 lymphatic vessel endothelial hyaluronan receptor-1, *MCAM* melanoma cell adhesion molecule, *PECAM*−1 platelet endothelial cell adhesion molecule-1, *TEM*8 tumor endothelial marker 8, *TGF*-*β* transforming grow factor-β, *Tie*−2 angiopoietin-1 receptor, *TNAP* tissue-nonspecific alkaline phosphatase, *TNF*-*α* tumor necrosis factor-α, *VCAM*−1 vascular cell adhesion molecule-1, *VE*-cadherin vascular endothelial-cadherin, *VE*-*statin* vascular endothelial-statin, *VEGFR*2 vascular endothelial growth factor receptor 2, *VEGFR*3 vascular endothelial growth factor receptor 3, *VWF* von Willebrand factor

**Fig. 1:**
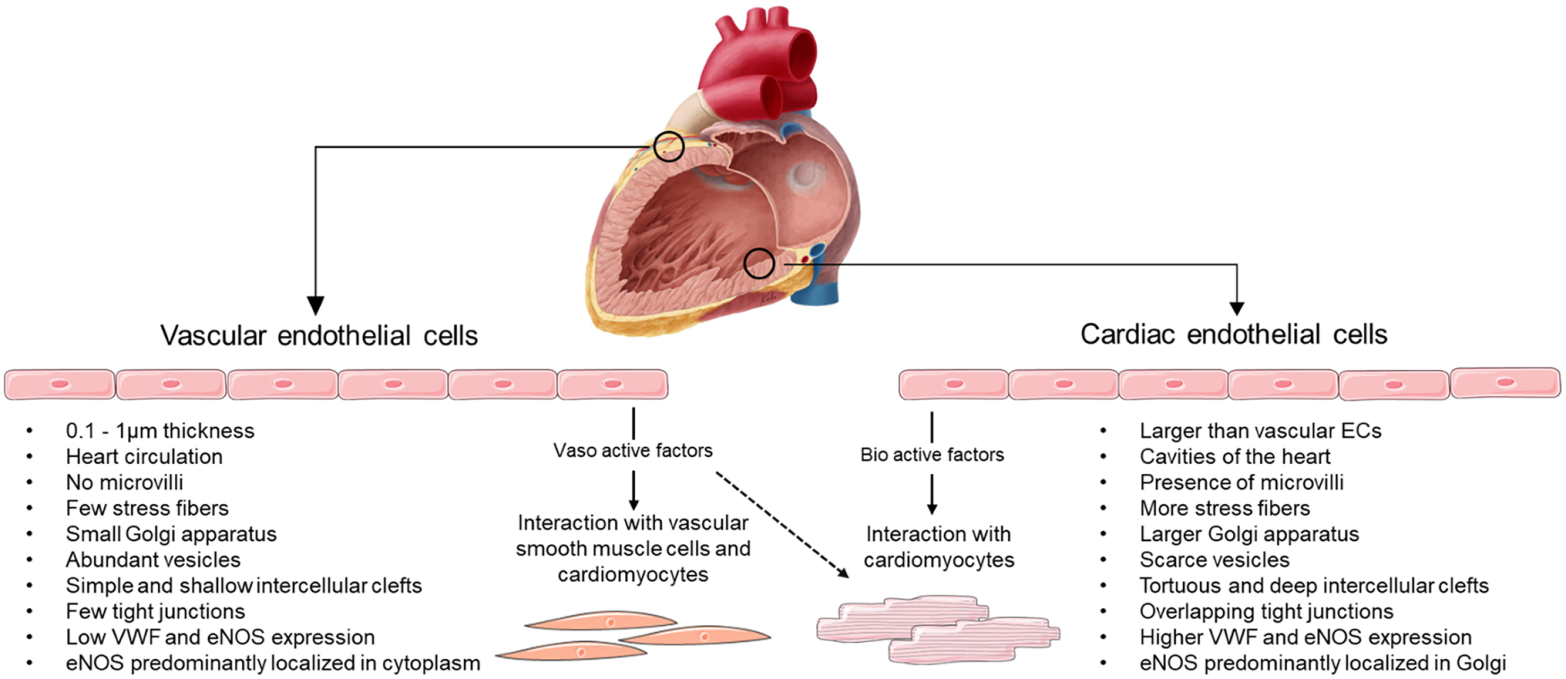
Characteristics of vascular and cardiac endothelial cells. Created with Smart Servier Medical Art (https://smart.servier.com/)

In the human heart, cardiomyocytes occupy approximately 70 to 85% of the cardiac volume [19]. However, ECs also have a pivotal role in the development and maintenance of cardiac homeostasis. ECs in the heart are classified based on their anatomical location, determining their direct or indirect effect on other cardiac cells. Thus, these cells can make part of the cardiac endothelium (ECs from the endocardium) and the vascular endothelium (ECs from the internal cellular layer of cardiac vessels) (Fig. 1) [20]. The cardiac endothelium and the cardiomyocytes share the same embryological origin, which is the cardiogenic mesoderm [21, 22]. It is the active crosstalk between ECs, vascular smooth muscle cells, fibroblasts, and cardiomyocytes that enables heart development, regulation, and adaptation [23]. ECs from the cardiac endothelium form the endocardium, delineating the inner lining of the heart chambers [22, 24]. The endocardium is composed of more strongly bound, often overlapping, ECs, due to a higher number of tight junctions that are critical to form a blood–heart barrier and maintain cardiac rhythmicity and mechanical performance [20].

These ECs are larger than other types of ECs (EC thickness varies from less than 0.1 μm in capillaries and veins to 1 μm in the aorta) [25], showing an increased surface area due to the presence of microvilli and trabeculae, and form deeper intercellular clefts than vascular ECs [22]. As the cardiac endothelium occupies a large surface area in the cardiac chambers, it is exposed to all elements circulating in the blood [20, 26]. It can sense biochemical changes and act as a paracrine regulator of cardiomyocyte function, secreting nitric oxide (NO), endothelin, and prostacyclin [26]. Moreover, VWF [27] and endothelial NO synthase (eNOS) [28] expression is also higher in the cardiac endothelium. eNOS is highly concentrated in the Golgi apparatus, whereas it is predominantly diffused in the cytoplasm in myocardial capillary ECs [29].

In terms of the cytoskeleton, ECs from the endocardium contain more microtubules, stress fibers, and filamentous vimentin networks than vascular ECs, being more closely packed [24]. Cardiac ECs also have a larger Golgi apparatus, fewer vesicles, and higher metabolic activity than vascular ECs [20, 24].

Moreover, cardiac endothelium plays a crucial role in fatty acid uptake for cardiomyocytes, whose main energetic pathway is mitochondrial fatty acid oxidation [30]. In fact, it has been shown that genes that regulate fatty acid uptake, such as the *Meox2/Tcf15*, *Fabp4*, and *Cd36,*, are highly upregulated in mice cardiac ECs [31]. ECs from the vascular endothelium (from both the macro and microcirculation) produce ATP mainly by glycolysis [32].

The ECs of the coronary arteries originate from mesoderm-derived proepicardium [33] and, in general, are similar in structure and function to other arteries in the body [22]. The coronary arteries originate from the aorta and penetrate the myocardium. A successive branching ultimately gives origin to a capillary network that surrounds the cardiomyocytes [22]—the myocardial microvessels. These present a continuous endothelium that is in intimate contact with the cardiomyocytes (only 1 µm apart), allowing for direct cellular communication and signaling, and thus regulating their function [20, 34]. This proximity is also optimal for oxygen (O_2_) and nutrient diffusion between the blood and the myocardium [22]. The vascular endothelium indirectly controls cardiac function by adjusting the perfusion of the myocardium [35].

The interaction between vascular ECs and vascular smooth muscle cells occurs directly, through main crosstalk mechanisms: paracrine regulation by bioactive molecules, communication via gap junctions, or information transfer via extracellular vesicles or extracellular matrix (ECM) [36].

In general, ECs are connected side-by-side through transmembrane adhesion proteins [37], including vascular endothelial and neural cadherin at adherent junctions [38, 39], occludin [40], and members of the claudin family [41], as well as the junctional adhesion molecule (JAM) family at tight junctions [42]. This cell-to-cell adhesion is translated into intracellular signaling pathways [37], culminating in specific alterations within the cell, at the cytoplasmatic level. Even though the molecular processes are not yet entirely understood [37], it is known that the signaling cascades triggered by intercellular junctions control key processes such as cell growth and apoptosis [43, 44]. The activation of these junctions may also induce long-term structural changes, such as the formation of tight associations with pericytes (which may encompass gene expression modulation), determining the stability and maturation of the vasculature [45], or short-lasting effects, like transient changes in endothelial permeability to solutes and circulating cells [46–48]. A further understanding of how the endothelial junction organization works is of paramount importance to decipher how ECs sense environmental changes.

Being capable of responding to environmental changes, ECs represent the main regulators of vascular homeostasis [3]. ECs reveal differences at the gene expression level, surface antigens, and morphology depending on their state—physiological or pathological [9]. A healthy endothelium is capable of responding to physical and chemical signals by the production of several factors that regulate vascular tone and cellular adhesion [49]. Moreover, it can inhibit the adhesion of leukocytes and platelets to the vessel walls [50, 51], contributing to preventing thrombus formation. The endothelium can also regulate inflammation and vascular smooth muscle cell proliferation [49]. The regulation of the vascular tone is a major function of the endothelium. ECs can produce and respond to vasoactive mediators, responsible for vessel relaxation or constriction. Each mediator balances tissue O_2_ supply by altering vessel tone and diameter and can contribute to a long-term remodeling of vascular structure, subsequently governing organ perfusion [52]. A well-known endothelium-derived relaxing factor is NO [53], generated from L-arginine by the action of eNOS [54]. Under physiological conditions, laminar blood flow keeps the vascular wall in a quiescent state, with a predominance of NO signaling, which inhibits inflammation, cellular proliferation, and thrombosis [55, 56]. NO is responsible for cyclic guanosine monophosphate (cGMP)-mediated vasodilation in vascular smooth muscle cells, controlling the adaptation of organ perfusion to changes in cardiac output [49]. ECs also mediate vasoconstriction via endothelin (ET) and vasoconstrictor prostanoids [57]. ET is a well-recognized vasoconstrictor, with ET-1 isoform being expressed in vascular ECs [58]. While the production and release of ET-1 are stimulated by inflammatory cells, its decrease is mediated by NO and prostacyclin [58]. Released ET-1 acts autocrinally [59] and paracrinally on other ECs and vascular smooth muscle cells [60, 61]. Prostacyclin and thromboxane act synergistically [62] and are synthesized by cyclooxygenase 1 (expressed continuously in ECs) and 2 (expressed when the endothelium is damaged) [63, 64]. Prostacyclin binds to its receptor in vascular smooth muscle cells and platelets [65], promoting relaxation of the smooth muscle [66] and inhibiting platelet aggregation [67], respectively. When NO is blocked, prostacyclin plays a compensatory role in vessel dilation [68]. Thromboxane, in turn, causes platelet aggregation and vasoconstriction [69]. In addition, ECs are capable of communicating with immune cells [70]. In hypoxic conditions, ECs initiate a pro-angiogenic response that causes loss of barrier function and facilitates cardiac immune cell infiltration and subsequent inflammation and edema [71, 72]. Additionally, through EC-secreted factors (chemoattractant and adhesion molecules), ECs enhance leukocyte infiltration. If not resolved, the continuous immune cell accumulation will be detrimental to an already damaged cardiac tissue [72].

## Endothelial cell dysfunction in cardiovascular diseases

**Fig. 2:**
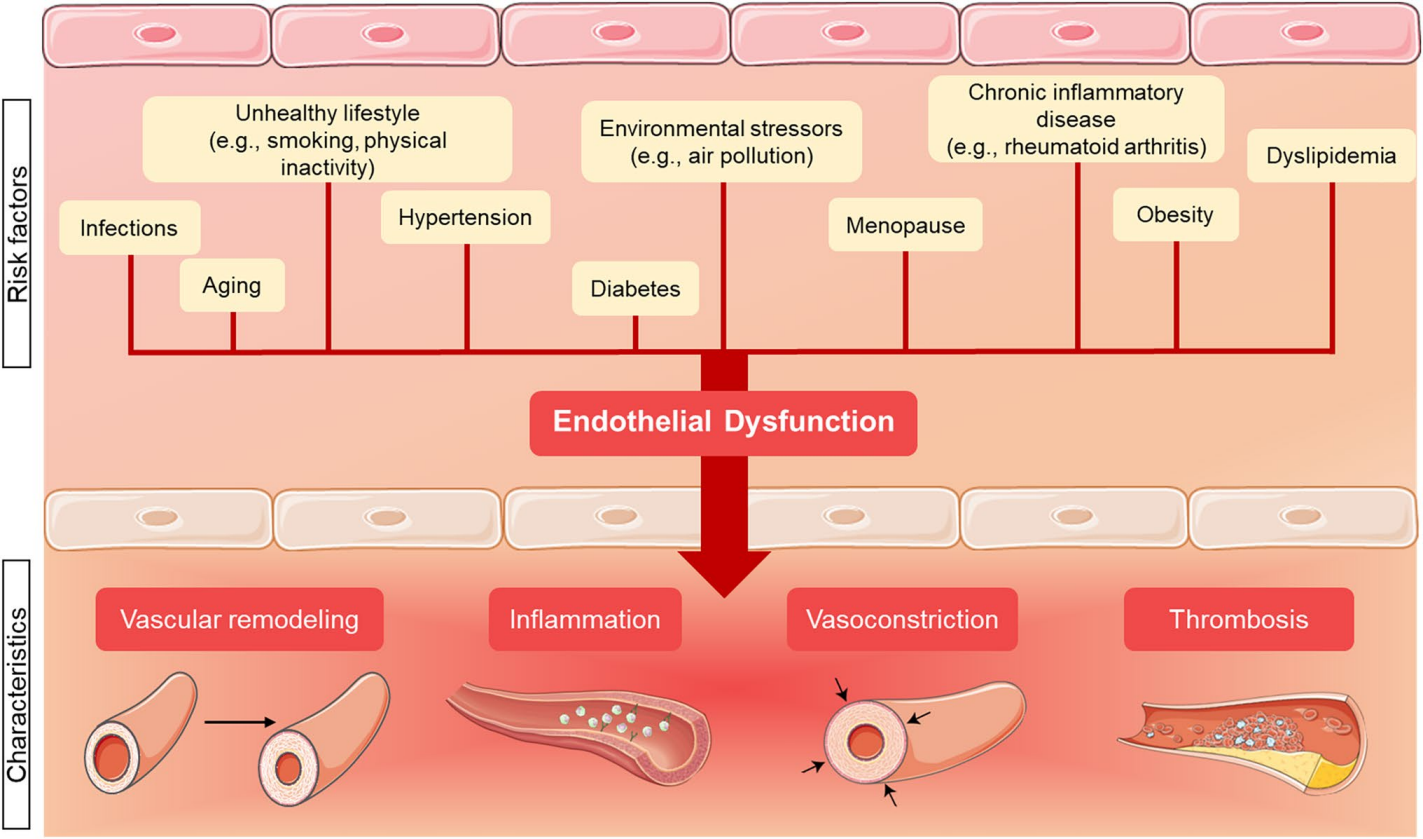
Risk factors for endothelial cell dysfunction and its pathological consequences. Created with Smart Servier Medical Art (https://smart.servier.com/)

In healthy arteries, ECs remain typically in a quiescent state, supported by, among other factors, a laminar blood flow. Nevertheless, various stimuli such as chronic disease states, metabolic conditions (e.g., type 2 diabetes mellitus [T2DM], obesity, dyslipidemia), smoking, and disturbed blood flow can disrupt such a quiescent phenotype and lead to endothelial cell dysfunction (ECD) (Fig. 2).

Different conditions, such as hypertension, atherosclerosis, and heart failure, are associated with varying endothelial cell phenotypes [73]. Until now, no universal definition of ECD has been recognized. However, the definition proposed by Segers et al. seems to be broad enough to capture most of its aspects. They suggested the following definition: “ECD represents all pathophysiological changes in endothelial cells related to disease; these changes include genetic, epigenetic, transcriptomic, proteomic, metabolic, morphological, and functional changes” [74].

To better understand the mechanisms driving ECD, it is crucial to examine the various triggers that disrupt the quiescent state of ECs. Factors such as inflammation, oxidative stress, hyperglycemia, hypoxia, toxins, and shear stress each play a significant role in altering ECs function and contributing to disease.

## In vitro models of endothelial cell dysfunction

**Fig. 3:**
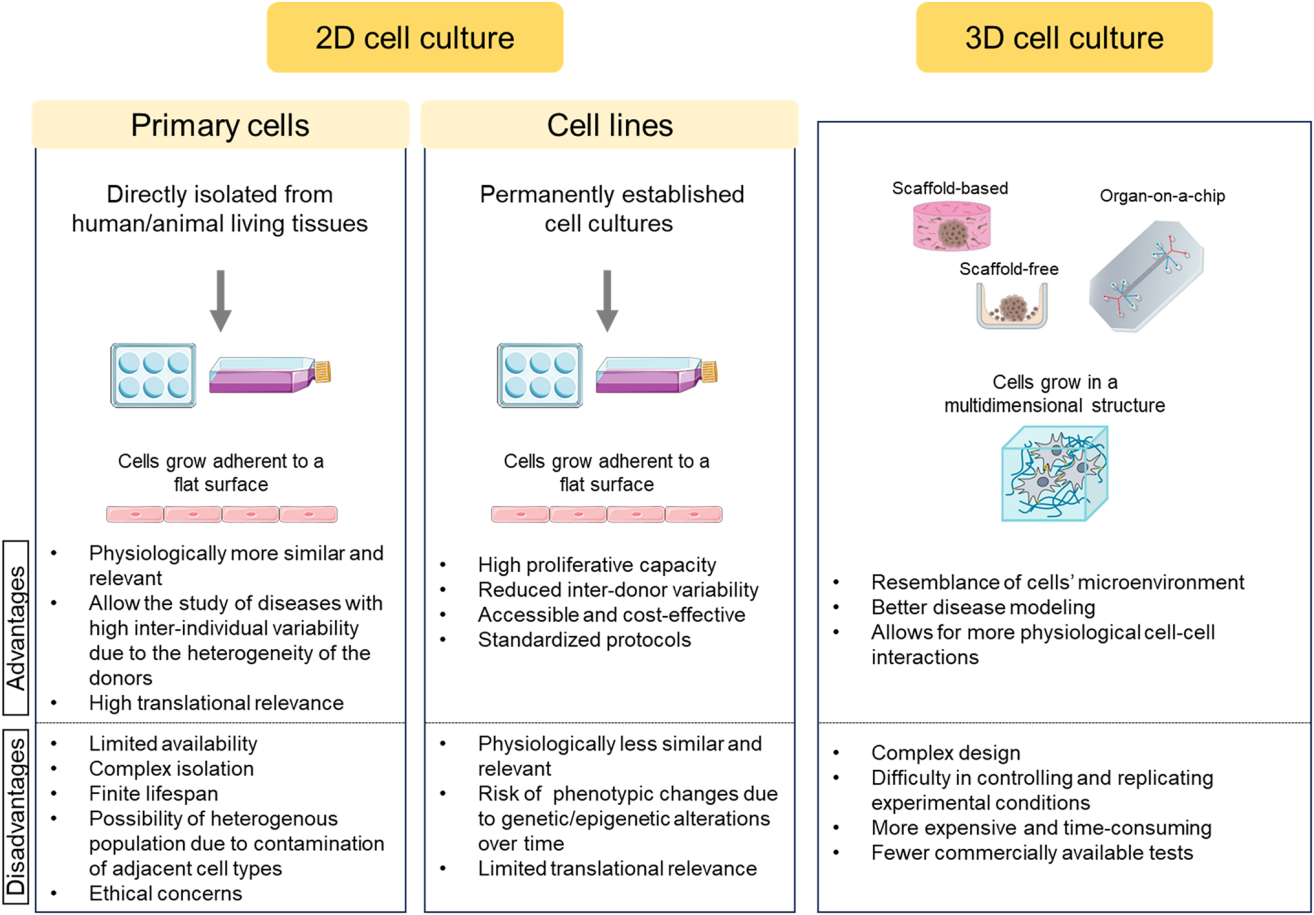
Types of cell culture models—2D and 3D—and respective advantages and disadvantages. Created with Smart Servier Medical Art (https://smart.servier.com/)

The complexity of ECD, associated with the distinct triggers that can provoke it, has been untangled with the utilization of several in vitro models. Different stimuli, chemical or mechanical, may be applied to ECs in culture, depending on the required outcome in terms of cellular dysfunction. These stimuli can be applied to 2D or 3D culture models (Fig. 3).

The shape of ECs varies in different in vitro models. In the standard 2D cell cultures, cells reveal a cobblestone shape, whereas in more advanced models with a dynamic flow, cells mimic the physiologic shape of vascular ECs in vivo, acquiring an elongated shape, due to shear stress [75].

Cell lines can be propagated repeatedly and sometimes indefinitely [76]. The use of cell lines in culture offers a consistent and uniform population of cells, reducing variability in results compared to primary cell cultures [77, 78]. Moreover, as nowadays cell lines are broadly used, they are readily available from cell banks, which makes them more easily accessible, and more cost-effective [77]. However, phenotypic changes are poorly controlled in cell lines and tend to appear over time due to the accumulation of mutations [78]. Primary ECs, in turn, are sourced either from animal tissues or patient biopsies and offer the possibility of comparing a diseased phenotype and a dysfunctional status to control cells [79, 80]. Primary cell cultures reveal greater heterogeneity, and their inherent limited replication potential necessarily implies the preservation of the in vivo characteristics, providing a more physiological model [81]. However, protocols for cell isolation and the establishment of primary cell cultures may be technically challenging, costly, and time-consuming [81]. HUVECs have the particularity of exhibiting in vitro behavior akin to cells in vivo [82]. For this reason, they are recognized as a model system for investigating the effect of disease triggers such as hyperglycemia, oxidative stress, and hypoxia on the endothelium, as well as angiogenesis, and cellular immune responses mediated by leukocytes [83–87]. Even though they can be commercially acquired as a cell line, being readily characterized and accessible, one disadvantage lies in the fact that they may not express all the surface molecules as primary cells do, due to the immortalization process, making them a less reliable model of the in vivo behavior [88]. 3D cell culture models are advanced in vitro systems designed to mimic the complexity of human tissues and organs better than traditional 2D cell cultures. These models are capable of more effectively recreating the multicellular architecture and cell-to-cell interactions, being a more reliable and versatile technique for the study of several complex mechanisms of disease [89]. Therefore, when we intend to do an experiment to study ECD using cell culture, the source of the cells and the type of in vitro model to be used must be well planned since there are various options available (Fig. 3), each one embracing specific advantages and limitations.

### Chemical stimuli

A great variety of in vitro models of ECD has been established by applying a chemical stimulus to cell lines or primary cell cultures. Next, we review models induced by different chemical stimuli.

#### Vasoconstriction/vasodilation imbalance

The induction of an imbalance in the availability of vasoconstrictors and vasodilators may promote ECD. A recent study tested the reduction of NO synthesis as a stimulus to mimic ECD [90]. This study was carried out in the murine thymic endothelioma cell line (tEnd.1), which has been proven to maintain the functional properties of normal endothelium [91, 92]. Additionally, tEnd.1 has a high NOS activity [93] and a 200-fold higher NO synthesis than the untransformed strains [94]. Cells were treated with different concentrations (from 1 to 1000 µM) of a NO synthesis inhibitor—L-NAME—during different times (12, 24, 48, 72, 96, and 120 h), with and without retreatment every 24 h (retreatment was applied to approximate this in vitro model to the chronic exposure that occurs in vivo). This study proposes L-NAME as capable of inducing ECD, showing that the conditions of 100 μM L-NAME for 72 h without retreatment and 100 μM L-NAME for 96 h with retreatment were the most effective. However, the concentration of L-NAME and the time of exposure may need some adaptations depending on the research interest and the type of cells in use.

Instead of vasodilation inhibition, in other models, vasoconstriction is induced with angiotensin II, ET-1, or thromboxane A2, to promote ECD. For instance, human microvascular ECs (MVECs) were incubated with Ang II (200 nM) during 48 h, showing ECD and vascular remodeling via downregulation of the ion channel TRPV4/eNOS pathway [95]. Moreover, in isolated rat cardiac MVECs treated with Ang II (4 nM) during 48 h, an induction of apoptosis was observed via regulation of the protein tyrosine phosphatase 1B/PI3K/Akt pathway [96]. HUVECs were treated with ET-1 for 24 h and the endothelial microvesicles (EMVs) released into the supernatant from these cells were isolated. HUVECs treated with ET-1-generated EMVs showed a higher release of interleukin (IL)−6, IL-8, total NF-κB p65, and active NF-κB p65 expression than HUVECs treated with control EMVs. Total eNOS and activated eNOS were significantly lower in HUVECs treated with ET-1-generated EMVs compared with control EMVs. Thus, ET-1 induced an EMVs phenotype that negatively affects endothelial cell function [97]. Thromboxane A2 has been associated with different cardiovascular diseases by its effects on inducing, for instance, vasoconstriction. Thus, this mediator can be used in a model of ECD. Human MVECs treated with a thromboxane A2 mimetic at 1 µM increased the levels of IL-8 mRNA expression and protein secretion in a time-dependent manner. IL-8 is a main inducer of endothelial permeability, and its increase is observed early in angiogenesis, besides being a hallmark of chronic inflammation in atherosclerosis, or diabetic retinopathy [98].

#### Oxidative stress

Oxidative stress is a pivotal mechanism behind ECD [99, 100], consisting of an imbalance of the redox state due to reactive oxygen species (ROS) overproduction, antioxidant systems depletion, or both; hence, models related to the increase of pro-oxidants have been explored. ROS can lead to hydrogen peroxide (H_2_O_2_) generation (in the presence of superoxide dismutase), which diffuses within the cell, oxidizing and altering protein functions [101], resulting in deleterious consequences. Although endothelial activation and redox signaling compose a normal host defense, when this activation is sustained, chronic production of ROS may exceed the capacity of cellular enzymatic and nonenzymatic antioxidant defenses [49], contributing to triggering vascular diseases such as atherosclerosis or peripheral artery disease [102, 103].

ROS can reduce endothelial barrier function, facilitating lipoprotein deposition and oxidative modification of low-density lipoprotein (LDL) particles in the vessel wall [104]. The role of oxidized LDL (Ox-LDL) particles as mediators of ECD is well established in the development of many CVDs, such as atherosclerosis [105]. Atherosclerosis starts with the accumulation of lipoproteins and inflammatory cells within the vascular wall. The disease progresses with the formation of fibrofatty lesions in the walls of arteries, leading to adverse health events such as myocardial infarction, stroke, and peripheral artery disease [106]. By interacting with lectin-type Ox-LDL receptor-1, Ox-LDL is capable of suppressing the constitutive eNOS expression [107], elevating the expression of adhesion molecules and chemokines in ECs, and inducing macrophage proliferation, collagen production, vascular smooth muscle cell migration, and platelet activation [108]. The continued stimulation by Ox-LDL impacts the expression of cellular tight junction proteins (occludin) and the endothelial monolayer becomes hyperpermeable [109, 110], aggravating ECD.

Ox-LDL particles have been used to set in vitro models of ECD. Two in vitro models have already been described [111, 112]. Both models exposed HUVECs to the same dosage of Ox-LDL, 100 μg/mL. However, they differed on the exposure time: 24 h [111] or 48 h [112]. In the first model, ECD was confirmed by the significant increase of the endothelial monolayer permeability and damage of tight junctions [111]. The second one further reported an antiproliferative behavior of HUVECs, limited migration, and loss of endothelial barrier function. The apoptosis rate was significantly increased, as well as the release of inflammatory factors—IL-1β and tumor necrosis factor (TNF)-α [112]. Both models using Ox-LDL are suitable in vitro models of ECD and could be particularly helpful for the study of atherosclerosis.

Also, increased ROS production can be attributed to the activity of the pro-oxidant enzymes NADPH oxidases (NOXs) [113]. NOX5, specifically, is regulated by intracellular calcium (Ca^2+^) levels, but also by CVDs-related stimuli, such as angiotensin II and ET-1 [114]. The human brain microvascular endothelial cell line—hCMEC/D3 was used to set a model of ECD by NOX5 overexpression [115]. A recombinant adenovirus codifying for human NOX5-β cDNA was used to induce NOX5 overexpression in ECs. This model using hCMEC/D3 favored ECD, by decreasing cell proliferation, increasing caspase 3/7 levels, inducing apoptosis, increasing oxidative phosphorylation, inducing mitochondrial dysfunction, and promoting higher cell migration [115].

In HUVECs treated for 1 h with different concentrations (200, 400, 600, 800, and 1000 µM) of H_2_O_2_, cells showed reduced cell viability, migration, and angiogenic capacity, along with a significant increase in senescence markers (β-galactosidase) [116].

#### Inflammation

The endothelium’s initial reaction to different inflammatory factors, known as endothelial activation, plays a crucial role in triggering a series of events that ultimately result in dysfunction [117]. For instance, during the early phases of acute myocardial infarction, the acute onset of high-grade local inflammation is revealed not only by the polymorphonuclear neutrophils activation and recruitment of mononuclear cells but also by the local release of pro-inflammatory molecules such as IL-1 and IL-6 [118]. These pro-inflammatory cytokines activate neutrophils, which release myeloperoxidase and catalase, giving way to an oxidative burst with implications on local tissue damage and initiating ECs junctional disassembly [118–120]. At the site of infarction, the endothelial adherent junctions are disrupted by ROS, cytokines, chemokines, thrombin, histamine, platelet-activating factor, vascular endothelial growth factor, and bradykinins [119]. This disruption is responsible for the increase in microvascular permeability and local post-infarction edematous state [121]. As mentioned, ECs are of paramount importance in regulating cardiomyocyte function given their location in the inner lining of blood vessels (especially in the cardiac microvascular endothelium) and endocardial epithelium [20, 122]. A link between microvascular and cardiac function following an ischemia–reperfusion injury has been reported [123]. The preservation of endothelial function, as seen in isolated rat hearts treated with verapamil (an L-type calcium antagonist used in the treatment of hypertension and stable angina), correlates with improved coronary flow, functional recovery, and reduced histopathological markers in ECs [123]. This underscores the role of endothelial cell-induced vasodilation in myocardial function after an ischemia–reperfusion injury. When a generalized and severe stage of inflammation is achieved, patients may develop sepsis, a life-threatening condition [124]. It has been proposed that sepsis may also accelerate pre-existing cardiovascular dysfunction/disease [125].

In vitro models of inflammation associated with ECD have been established using various triggers, such as lipopolysaccharide (LPS), interferon γ (IFNγ), and TNF-α. As an example, HUVECs were treated with 50 ng/mL IFNγ, followed by LPS (0, 10, 100, or 1000 ng/mL) [126]. With LPS treatment only, endothelial permeability increased and there was a significant loss of the glycocalyx. The endothelial glycocalyx is a gel-like layer that lines the luminal surface of blood vessels, primarily composed of proteoglycans, glycoproteins, and plasma proteins [127]. It functions as a protective vascular barrier, regulating vascular permeability and mechanotransduction, while also preventing leukocyte adhesion and platelet aggregation to maintain vascular homeostasis [128]. The loss of glycocalyx is closely associated with the development of ECD and is strongly influenced by the degree of inflammation. Acute inflammation, as seen in sepsis or ischemia–reperfusion injury, leads to rapid and extensive glycocalyx degradation, while chronic inflammation, as observed in diabetes, hypertension, and atherosclerosis, results in progressive but less severe degradation [128, 129]. However, chronic injury impairs the synthesis of glycocalyx components and alters their structure, making the glycocalyx more susceptible to external stressors [128]. When cells were treated with IFNγ only, a smaller, non-significant increase in endothelial permeability was noted and the loss of glycocalyx was non-significant. The combined effect of LPS and IFNγ revealed the largest increase in endothelial permeability and there was a significant loss of the glycocalyx, accompanied by a further decrease in heparan sulfate proteoglycan 2. Thus, LPS treatment resulted in significant increases in permeability and glycocalyx loss, IFNγ alone had minimal effects, while the combination of both treatments led to the most substantial increases in permeability and glycocalyx loss [126]. Also, in primary HUVECs, treatment with TNF-α (10 ng/mL) during 16 h induced CXCL1 release in the culture medium, and the same was found with treatment with LPS (100 ng/mL) [130].

#### Hypoxia

Chronic persistent or intermittent hypoxia (IH) triggers ECD leading to both inflammation and oxidative stress [131, 132]. Chronic persistent hypoxia is recurrent in chronic lung diseases where O_2_ levels remain constantly low, while chronic IH is characteristic of obstructive sleep apnea, characterized by an intermittent pattern of episodes of hypoxia and reoxygenation [133]. Both types of hypoxia are present in conditions that are risk factors for CVDs; however, evidence from animal [134, 135] and cellular studies [136–138] has demonstrated that chronic IH is more likely to cause ECD than chronic persistent hypoxia. At the cellular level, under hypoxic conditions, the hypoxia‐inducible factor (HIF)‐prolyl hydroxylase family of enzymes (PHD1‐3) lose their ability to hydroxylate HIF because they rely on O_2_ for their enzymatic activity [139]. In normoxia, hydroxylation of HIF-α by PHDs enables its interaction with pVHL (von Hippel-Lindau protein) and targets α subunits for ubiquitination and proteasomal degradation [140]. Under hypoxic conditions, the inhibition of PHDs activity results in HIF-α accumulation and translocation to the nucleus, which, together with the HIF-β subunit forms, can activate transcription factors (HIF-1, HIF2, or HIF-3) [141]. As a result, several genes are regulated to counteract the impacts of hypoxia, promoting, for instance, angiogenesis and glycolysis, while also leading to the onset of pathological processes [142, 143]. For example, chronic IH dysregulates the expression and activity of eNOS, disrupting NO production and, thus, contributing to the development of ECD. It has been shown that eNOS is downregulated by hypoxia in vitro, in HUVECs [144, 145], human coronary artery ECs (HCAECs) [146], and bovine pulmonary artery ECs (PAECs) [147]. In addition to changes in eNOS expression, hypoxia can also result in post-translational modifications of eNOS that change its activity. In hypoxic HUVECs, eNOS Ser1177 phosphorylation was shown to be reduced, whereas Thr495 phosphorylation was increased, resulting in altered eNOS activity and reduced NO production [148]. Chronic IH may also result in an imbalance in the ratio of arginase-1 (an enzyme that competes with eNOS for L-arginine) to eNOS expression, resulting in decreased NO availability, a key feature of ECD [149]. Chronic IH was found to result in selective activation of inflammatory pathways mediated by the transcription factor NF-κB rather than adaptive pathways dependent on HIF-1 both in HeLa cells [150] and human endothelial EA.hy926 cells [151]. The pro-inflammatory cytokines (TNF-α, IL-6, IL-8, E-selectin) and adhesion molecules (VCAM-1 and ICAM-1) stimulated by NF-κB are implicated in the development of ECD [152, 153]. Chronic IH has also been found to result in increased ROS production, altering ECs function. For example, in pulmonary MVECs exposed to chronic IH, ROS production increased and was associated with endothelial barrier dysfunction due to ROS-dependent activation of extracellular signal-regulated kinases (ERK) 1/2 and c-Jun N-terminal kinases that initiate the reorganization of cytoskeletal and junctional proteins [154]. In HUVECs under hypoxic conditions, ROS production also increased and impaired microtubular structure via the PI3K/Stathmin 1 pathway [155]. Chronic IH can also result in an imbalance between pro- and antioxidant systems, ultimately leading to excessive ROS production and oxidative stress in ECs [156]. For example, in human PAECs under hypoxic conditions, the levels of ROS were increased, while the levels of mitochondrial thioredoxin 2, an antioxidant protein, were reduced which could contribute to exacerbating hypoxia-induced ROS production [157].

Different frequencies and degrees of hypoxia have also been used as in vitro models of ECD [136]. For example, EA.hy926 cells (produced by hybridization of HUVECs with the epithelial cell line A549) were housed in a customized chamber where premixed air was delivered in a controlled way. Several cycles of IH and reoxygenation were programmed, resulting in cyclic fluctuations of cellular O_2_ pressure. Cells were divided into six experimental groups (IH1 to IH6). All groups were exposed for 5 h to 1.5% O_2_ concentration levels, except for IH6, which received 10% at the same frequency. In groups IH1 to IH5, the duration of IH, as well as the O_2_ concentration, was maintained stable, while reoxygenation was progressively shortened, and the frequencies gradually increased. The authors concluded that the inflammatory responses, oxidative stress, and imbalance of vasoactive substances caused by IH should be related to the frequency and degree of hypoxia. mRNA expression of NF-κB p65, TNF-α, and c-fos (a constituent part of the activator protein complex-1, a transcription factor associated with inflammation [158]) did not increase gradually with the increase in frequency. Instead, they increased from the IH1 to IH3 groups and then decreased gradually in IH4 and IH5 groups. Notably, the duration of reoxygenation emerges as a critical factor influencing ECD. However, a longer duration of reoxygenation was not associated with more severe damage to the cells. When the IH frequencies were further increased, the reoxygenation cycles were necessarily shortened, and ECD was moderated. However, when the reoxygenation was extended for longer, cell repair started and ECD decreased [136]. A higher degree of inflammation was verified in groups IH1 to IH3 (IH period of 1.5% O_2_ for 15 s followed by reoxygenation periods of 21% O_2_ for 8 min and 15 s, 5 min and 15 s, and 3 min and 45 s, respectively). A higher level of oxidative stress and a major imbalance of vasoactive substances (NO and ET-1) was verified in groups IH3 and IH6 (IH6: IH period of 10% O_2_ for 15 s followed by a reoxygenation period of 21% O_2_ for 3 min and 45 s) [136]. In conclusion, the key phase to induce different effects of ECD with this model is the duration of the reoxygenation cycle.

#### Hyperglycemia

Diabetes is associated with the accelerated disappearance of capillary endothelium [159], morphological and functional alterations of ECs [160], and the weakening of intercellular junctions [161], which ultimately leads to ECD. In diabetes, when protective stimuli such as acetylcholine [162] and insulin [163] are diminished, there is a decrease in eNOS activity and expression, as well as eNOS uncoupling. This results in reduced NO production and increased production of radical superoxide [164]. Hyperglycemic conditions not only reduce NO production but also enhance NF-κB activation and promote inflammatory gene expression and leukocyte recruitment [165]. NF-κB is a key transcriptional regulator of inflammatory mediators and leads to, for example, increased expression of TNF-α, IL-6, IL-8, E-selectin, VCAM-1, and ICAM-1 [166–168]. Even transient hyperglycemia has been shown to induce epigenetic modifications that sustain NF-κB expression and promote ECD even after the restoration of normoglycemia [169]. Hyperglycemia instigates the formation of advanced glycation end products (AGEs), irreversibly modifying proteins, therefore contributing to ECD [170]. AGEs interact with cell surface receptors like RAGE, triggering endothelial ROS production and pro-inflammatory gene expression [171]. Additionally, AGEs reduce NO production and inhibit histamine-induced NO production in ECs [172]. AGEs also modify proteins from the ECM, leading to vessel stiffening and ECs stress [173–177]. In the setting of hyperglycemia, there are several sources of ROS production, such as mitochondrial superoxide production, eNOS uncoupling, and AGE-dependent NOXs activation [178–180]. In summary, the hyperglycemic state inherent to diabetes leads to ECD and inflammatory responses that persist even after normoglycemia restoration, due to epigenetic modifications [175]. While antioxidant therapies may show promise in mitigating oxidative stress and inflammation in experimental models, there is still a large number of inconsistent clinical outcomes given the complexity of targeting ROS-mediated pathways in diabetic vascular complications [181].

The sustained exposure of ECs to high glucose levels is another way to induce ECD. For instance, HUVECs were cultured with high glucose levels (30 mmol/L) for short-term (48 h and 72 h) and long-term exposures (13 days) [182], and both exposure times consistently induced cell apoptosis, showing approximately a 20% increase after 48 h and around a 46% increase after long-term exposure. Human retinal MVECs (HRMVECs) were also used for the study of diabetic retinopathy, a condition characterized by ECD [182]. HRMVECs were treated with increasing concentrations of D-glucose (25, 50, 100, and 150 mM), and the control group with mannitol in the same concentrations. It must be noted that the osmotic pressure adjustment in the control group should be made by adding mannitol or another suitable organic solute to the medium, because these are biocompatible and non-toxic, making it a suitable choice for adjusting osmotic pressure without interfering with the biological processes under study [183]. In this model, 25 mM and 50 mM glucose were deemed significant hyperglycemia thresholds to severely inhibit ECs proliferation. The proliferation of ECs was enhanced at 25 mM but diminished at 50 mM and 100 mM, while the migration capacity increased at 25 mM and 50 mM and decreased at 100 mM. Apoptosis was higher at 25 mM and 50 mM, and cell permeability increased with rising glucose concentrations, particularly at 48 h after treatment [182]. Additionally, VEGF-A secretion increased linearly with glucose concentration [182]. VEGF-A is known to promote the retinal expression of ICAM-1, promoting the adhesion of leukocytes to the retinal vessels, the disintegration of the blood–retina barrier, ECs damage, and apoptosis [184].

Another culture model was employed by Leng et al. [185], in which rat aortic endothelial cells (RAOECs) were isolated from the aorta of diabetic Sprague Dawley rats. RAOECs were cultured in a high glucose medium (33 mM glucose) for 48 h. After being exposed to hyperglycemia, the cells’ supernatant revealed an increase in cytokine levels—such as IL-6, TNF-α, IL1β, and IL-18. Besides, a decrease in glutathione peroxidase and superoxide dismutase activity was found in RAOECs, as well as an upregulation of the levels of ROS [185].

#### Toxins

Environmental stressors, such as air pollution [186], and numerous toxic substances [187] can lead to ECD. For instance, in HUVECs, incubation with arsenic (arsenite 20 µM) for 24 h induced a significant increase in AngII production in the cell supernatants and cell surface levels of AT1R. Also, the production of E-selectin, ICAM-1, IL-8, and MCP-1, evaluated in the cell culture supernatants, as well as the generation of ROS and malondialdehyde, in HUVECs, significantly increased with arsenic treatment [188]. Cigarette smoke is a factor known to induce ECD. In cultured bovine PAECs, cigarette smoke extract caused disruption of focal adhesion complexes, F-actin fibers, and adherens junctions and decreased activities of RhoA and focal adhesion kinase [189]. Also, p-cresol is a uremic toxin, found to accumulate in the serum of chronic kidney disease patients [190], revealing deleterious effects on the endothelium in vitro [191, 192], and inducing the formation of EMVs in cultured ECs [193, 194]. An in vitro model of ECD was described using p-cresol [195]. HUVECs were incubated with p-cresol at 25 μg/mL for 24 h, resulting in the generation of EMVs, which interfere with endothelial repair processes by decreasing the migratory capacity and the ability to form new vessels and by increasing cell senescence. All these cellular alterations are associated with ECD.

#### Nutrient deficiency

Cell starvation is another method used to cause ECD. For instance, to induce different degrees of starvation, the culture medium of HUVECs was changed at 48, 72, or 96 h, representing different degrees of starvation in comparison with non-starved cells (medium changed every 24 h). This model results in progressive nutrient constraints, seeking to be more physiological. The optimum time to induce ECD by starvation was 96 h since a decrease in angiogenic and migratory capabilities, as well as an increase in recognized markers of senescence, such as β-galactosidase, were observed [116]. Another in vitro model of ECD implies the treatment of ECs with low magnesium (Mg^2+^) concentrations. Low Mg^2+^ triggers a pro-oxidant and pro-inflammatory phenotype, retarding cell growth and promoting cell senescence [196–198]. Mg^2+^ is the fourth most abundant mineral in the human body [199], and its deficiency is one of the many factors that impair endothelial function [196, 199]. Physiologically, at the cellular level, Mg^2+^ is involved in metabolic pathways acting as an enzyme cofactor, contributes to the regulation of membrane stability, regulates ion channels, and acts as an intracellular signal [200, 201]. In HUVECs subjected to normal (1 mM) and low (0.1 mM) Mg^2+^ concentrations for 24 h, in a custom-made Mg^2+^-free medium supplemented with magnesium sulfate (MgSO_4_), Mg^2+^ deficiency upregulated the pro-oxidant protein thioredoxin-interacting protein (TXNIP), a contributor to ROS generation, increased endothelial permeability (altering inter-endothelial junctions by disrupting VE-cadherin distribution), and promoted the accumulation of triglycerides in lipid droplets [202]. The authors found that silencing TXNIP protein expression restored endothelial permeability and membrane localization of junctional proteins. Thus, in HUVECs, Mg^2+^ deficiency promotes oxidative stress, and the upregulation of TXNIP seems to participate in the accumulation of ROS, triglycerides in lipid droplets, and hyperpermeability [202].

### Mechanical stimuli

Vascular ECs, as integral components of blood vessels, are continuously exposed to mechanical forces generated by blood flow, including fluid shear stress and cyclic tensile stress [203]. These mechanical forces influence endothelial function and are crucial for capillary patterning, ensuring efficient oxygen and nutrient delivery [204]. They also drive vascular remodeling in major arteries, helping to maintain blood pressure, and regulate cell morphology, polarity and migration [204, 205]. These forces are primarily sensed through primary cilia, the glycocalyx, integrins, G-protein-coupled receptors, ion channels, CD31, VE-cadherin, and VEGFR2. However, the specific signaling pathways activated depend on the nature of the mechanical force applied, leading to distinct cellular responses.

Shear stress refers to the force exerted by blood flow parallel to the endothelial surface and can be classified as laminar flow and turbulent flow [203]. Laminar shear stress is a steady, unidirectional force exerted by blood flow in straight arteries and large vessels, promoting anti-inflammatory, anti-thrombotic, and vasoprotective effects. Activation of these sensors triggers PI3K/Akt signaling and eNOS activation, increasing NO production as well as promotion of nuclear factor erythroid 2-like 2 (Nrf2), responsible for antioxidant gene expression [206]. Moreover, MAPK/ERK signaling upregulates Kruppel-like factor-2 (KLF2) and KLF4, which enhance eNOS expression, inhibit NF-κB signaling, and suppress TNF-α receptor expression, reinforcing anti-inflammatory and atheroprotective effects [207]. Additionally, low laminar flow found in smaller vessels promotes a quiescent state in ECs, while in larger vessels, higher physiological shear stress induces ECs' elongation and alignment parallel to the flow direction [208, 209]. Also, the Golgi apparatus positions itself upstream of the nucleus, orienting against the flow direction, reflecting the establishment of front–rear polarity [210]. On the other hand, turbulent flow refers to the irregular, oscillatory mechanical forces created by disturbed blood flow at arterial branches, bends, and dysfunctional venous valves [206]. Unlike laminar shear stress, this type of shear stress is pro-atherogenic, pro-inflammatory and contributes to ECD, making it a key factor in the development of atherosclerosis and other cardiovascular diseases [203]. Turbulent flow promotes NF-κB and AP-1 activity that upregulate pro-inflammatory and pro-atherogenic genes such as ICAM-1, E-selectin, platelet-derived growth factor (PDGF)-BB, IL-1a, bone morphogenic protein-4 (BMP-4), monocyte chemotactic protein-1 (MCP-1), and ET-1 [211]. Additionally, under turbulent flow, ECs become randomly oriented, adopting a rounded or cobblestone-like morphology [212]. Cell–cell junctions weaken, leading to increased endothelial permeability, while polarity is disrupted, with ECs failing to align with the flow direction [209].

Cyclic stretch refers to the radial force caused by the stretching and relaxation of endothelial and vascular smooth muscle cells due to pulsatile blood pressure [213]. This mechanical stimulus can have both physiological and pathological effects on vascular function. Under physiological conditions, cyclic stretch plays a crucial role in maintaining vascular homeostasis. It activates the PI3K/Akt pathway, leading to eNOS activation and NO production, which promotes vasodilation and anti-inflammatory effects [214]. Additionally, it reduces ROS formation and limits apoptosis via the PI3K/Akt/Bad pathway [214, 215]. In contrast, pathological cyclic stretch, as seen in hypertension and atherosclerosis, contributes to ECD and cardiovascular disease progression [212]. It is linked with increased ROS production that reduces NO bioavailability impairing endothelial function [207, 214]. This pathological stretch also activates NF-κB, promoting the secretion of pro-inflammatory cytokines such as IL-6/8 and the expression of CD40 and VCAM-1, driving immune cell recruitment and contributing to atherosclerosis progression [214]. To model and study ECD induced by these mechanical stimuli, a variety of in vitro flow-loading and stretch loading devices have been developed. Next, we review some models induced by cyclic stretch and shear stress. This topic can be explored in more detail in [216, 217].

#### Cyclic stretch

Isolated cyclic stretch as a stimulus for ECD can be a valuable trigger [218]. However, it can also be useful in combination with other triggers, such as hypoxia, because studying multiple triggers provides a more holistic understanding of the mechanisms driving ECD and allows for a more robust and representative model that mirrors the pathophysiological conditions observed in vivo [219]. With this purpose, HUVECs were seeded into fibronectin-coated flexible silicone chambers to ensure optimal cell growth and adherence [220]. Then, cyclic stretch was applied by using a STREX® system. The STREX® system involves the application of controlled mechanical forces to biological samples (e.g., cells or tissues) with simultaneous monitoring of their responses, allowing for the precise and quantitative measurements of mechanical forces at the cellular level. Both physiological and pathological environments were mimicked, with variations in O_2_ levels (in both normoxic and hypoxic conditions) for 20 h to simulate cellular responses to mechanical and environmental stimuli [220]. Examining HUVECs morphology under various conditions revealed changes such as elongation and stress fiber formation [220]. Cyclic stretch and hypoxia synergistically increased ROS production, and both independently increased IL-6 secretion, nerve growth factor, and fibroblast growth factor-2 (FGF-2) secretion, with the latter two being implicated in endothelial cell proliferation.

The use of nanoparticles (NPs) could also be paired with cyclic stretch to recapitulate different physiological environments. In the work of Deweirdt et al. a model of endothelial cell culture was used to mimic the environment of pulmonary hypertension, combining both chemical and physical stimuli—NPs and cyclic stretch, respectively [220]. Exposure to inhaled NPs is thought to be an occupational hazard. However, its toxic effects on humans have not yet been widely recognized. Also, from an epidemiological standpoint, the impact of NPs on health remains uncertain [221, 222]. Briefly, human PAECs isolated from the main branch of the pulmonary artery of young male donors were cultured in fibronectin-coated (5 μg/cm^2^) flexible silicon chambers. Both physiological and pathological stimulations were performed. Physiological stimulation was done with static culture in normoxia (21% O_2_). In contrast, pathological stimulation involved cyclic stretch at 5% or 20% strain at 1 Hz frequency for 20 h in normoxia (21% O_2_) and in hypoxia (1% O_2_) combined with 20% cyclic stretch and exposure to NPs. After this initial culture and application of physical (e.g., stretch) and chemical (e.g., O_2_) conditions, the cells were exposed to FW2 NPs at different concentrations (5, 7.5, and 10 μg/cm^2^) for 4–6 h. The results showed that FW2 NPs significantly increased the production of ROS, and this was more pronounced under simulated disease conditions (hypoxia and cyclic stretch). Enhanced inflammatory status, marked by increased release of IL-6, was observed in cells exposed to FW2 NPs. Another similar model was employed by Germande et al. using human PAECs exposed to both nickel oxide NPs (NiONPs) and cyclic stretch [223]. A notable difference between the two models is the concentration of NPs, which were between 0.5 and 10 μg/cm^2^, and the treatment duration between 4 and 24 h. The results of NiONP exposure were similar to those of FW2 NPs, showing an increase in ROS production, release of IL-6, and altered Ca^2+^ signaling.

#### Shear stress

Another trigger of ECD is shear stress. Fluid shear stress was employed by Ghim et al*.* [224] in cultured cells in order to better understand the physical mechanisms underlying atherosclerosis. Shear stress was applied to HUVECs by placing plates on an orbital shaker for 3 days. After the cells were exposed to 48 h of shear stress, TNF-α was added for 24 h. The flow simulations used showed a varying shear stress profile across the well. When it comes to morphology, HUVECs in the annulus displayed an elongated phenotype, while those in the center exhibited cobblestone morphology. Nuclei elongation increased with radial distance under high-magnitude uniaxial flow. Monocyte adhesion was higher in the center compared to the edge of the well, particularly with TNF-α treatment. It was also found that segmentation increased monocyte adhesion and the expression of adhesion molecules in TNF-α-treated HUVECs at the center but not at the edge of the well [224]. In conclusion, the use of the cellular model presented by the work of Ghim et al*.* offers a view into the relationship between fluid shear stress and ECD. In order to enhance the applicability of this in vitro model, the effect of shear stress could be combined with other relevant triggers, such as hyperglycemia. This way, this model arises as a relevant tool for investigating the dynamics of vascular pathology. Other studies employed similar techniques, such as parallel-plate flow chamber systems (PPFCs), cone-and-plate systems, and orbital shaker systems to conduct various experiments with ECs [225–227]. Studies using PPFCs usually target the analysis of cell morphology and alignment in ECs such as porcine aortic ECs [228] and HUVECs [229], and showed that the cells align gradually with the flow direction, morphologically change from a cobblestone aspect to an elongated shape, and alter intercellular space properties such as the increase in junction length. Other investigations employing the cone-and-plate systems and orbital shaker systems in ECs experiments also analyze cell morphology [227, 230, 231], protein expression by Western blotting or ELISA (e.g., IL-6, FGF-2) [232–234], and mediators involved in eNOS activation [235, 236].

### 3D cell culture models

**Fig. 4:**
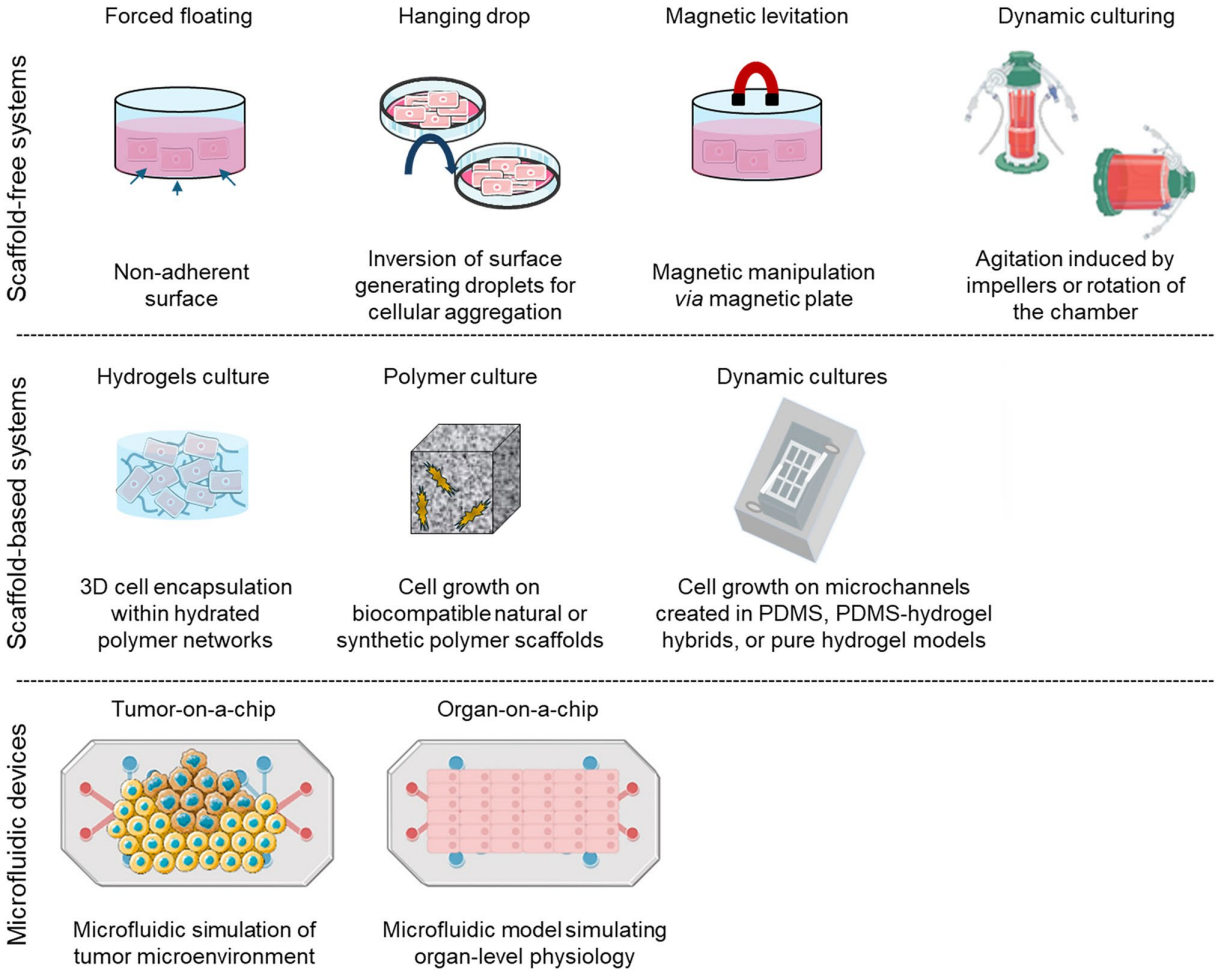
Types of 3D cell culture systems. Created with Smart Servier Medical Art (https://smart.servier.com/) and BioRender (https://www.biorender.com/)

Most in vitro studies of ECD rely on 2D cell models where a monolayer of ECs is grown on tissue culture polystyrene (TCPs) plates [237]. The relatively straightforward manufacturing process, low production cost, and optical transparency drive the mass production of this type of tissue culture plate, making it the primary platform for adherent cell cultures [238]. However, for numerous reasons, 2D models are very reductionist and oversimplify the in vivo conditions of ECs. For example, 2D cultures only allow interactions with neighboring cells and the formation of cell-ECM adhesions on the substrate side in the horizontal plane, whereas in 3D culture, cells are capable of forming adhesions on all sides in a dynamic way [239]. Additionally, in 2D cultures, ECs can easily spread and migrate due to the lack of physical impediments, while in 3D cultures, a surrounding matrix poses constraints that enforce the ECs’ adaptation to fit through matrix pores and often require matrix degradation for spreading and migration [240]. In fact, the vascular endothelium grows in the body as a monolayer of cells lining the inner surface of blood vessels within a 3D microenvironment supported by collagen and proteoglycan ECM [241, 242]. This microenvironment is of extreme importance due to its role not only as a structural, modifiable, and flexible scaffold for cells but also as a provider of a range of biochemical and biophysical signals that collectively regulate cell behaviors such as spreading, proliferation, migration, differentiation, and apoptosis [87]. In the case of ECs, the glycocalyx, as mentioned before, is a layer composed essentially of glycoproteins that reside on the surface of the cells and is the main element of their ventral microenvironment, in vivo [243]. However, 2D models fail to mimic the main ECM where ECs grow in vivo and the development of the glycocalyx is unknown in this type of culture [87]. Regarding ECD, especially in the context of CVDs, ECM remodeling is an important area of interest due to its relationship with alterations in the normal function of ECs. Current 2D models are very limited and physiologically inaccurate, and the data obtained from them may not translate to the in vivo setting, ultimately delaying the advancement of knowledge in this field. In fact, ECs cultured in 2D *vs.* 3D models have been shown to have different gene expression and morphological disparities [244]. The main goal of 3D cultures is to provide cells with a bulk ECM with properties that guide cells to develop or sustain a desired phenotype and, implicitly, to rely upon the cells themselves to create a local 3D microenvironment that mimics the in vivo microenvironment. Additionally, some 3D systems are specifically designed to incorporate hemodynamic forces, such as shear stress and shear strain from blood flow, which can significantly influence cell behavior [225]. Thus, current 3D cell culture systems are broadly classified into scaffold-based and scaffold-free, depending on whether cells grow with or without the support of a physical structure [245]. Furthermore, the incorporation of dynamic culture media flow within these models allows for an additional classification into static or dynamic 3D cell cultures. This section will explore these models in the context of ECD research, along with the recent emergence of complex microfluidic devices known as organ-on-a-chip systems (Fig. 4).

#### Scaffold-free 3D cell culture systems

In scaffold-free 3D cell culture systems, no exogenous matrix or support is used to mimic the ECM [246]. Instead, cell assembly relies on self-aggregation and the natural secretion of ECM components, with various cell–cell interactions driving the formation of 3D structures. The most straightforward method consists of culturing suspended cells in a nutrient medium, allowing their natural tendency to aggregate to form structured 3D cell clusters, known as spheroids [245]. Regarding static scaffold-free 3D cultures, the most common techniques employed are the forced floating method, the hanging drop method, and the magnetic levitation method while for dynamic systems, agitation-based techniques are applied using spinner flask bioreactors and rotational flask bioreactors [247]. These models are rarely used for culturing ECs alone to study ECD. Instead, they are more commonly applied to generate spheroids formed through the assembly of various cell types, a practice widely used in tumor research, but also with some applications in modeling cardiac tissue envisioning the potential use in treating CVDs [248–251]. For instance, co-culturing cardiomyocytes, ECs, smooth muscle cells, and cardiac fibroblasts derived from human-induced pluripotent stem cells (iPSCs) employing the forced floating method using ultra-low attachment plates have been shown to result in the formation of cardiac spheroids [252]. Researchers found that the spheroids composed of all four cell types demonstrated improved sarcomere maturation compared to spheroids containing only cardiomyocytes and highlighted the potential of this model for broader applications. Similar approaches have successfully generated cardiac spheroids using rat neonatal ventricular cardiomyocytes, human dermal fibroblasts, and human coronary microartery ECs in ultra-low attachment plates [253], as well as spheroids composed of human iPSC-derived cardiomyocytes, human cardiac fibroblasts, and HUVECs in an ultra-low attachment hanging drop system [254]. In both cases, the spheroids were engineered with the goal of serving as building blocks for cardiac tissue patches intended for future grafting into damaged native myocardium and aid in the treatment of heart failure. Additionally, a study successfully created magnetically ensembled spheroids composed of mouse myoblasts cells and HUVECs that had internalized magnetic nanoparticles (MNPs) extracted from *Magnetospirillum magneticum* [255]. Although these spheroids were initially designed for in vivo testing of their engraftment and vascularization potential in murine kidney and hindlimb ischemia models, the results showed that MNP internalization did not compromise HUVEC viability. Moreover, spheroids containing both cell types with MNP exhibited enhanced sprouting capability. Although these models were not originally developed to study ECD, they hold significant potential for such applications. By introducing known triggers of ECD into these 3D models, it would be possible to observe not only how ECs are directly affected, but also how their interactions with surrounding cell types are altered within the complex microenvironment of the spheroids.

#### Scaffold-based 3D cell culture systems

In scaffold-based 3D cell culture systems, ECM-like structures are introduced to provide physical support for cell adhesion, proliferation, and differentiation, typically in the form of polymer-based scaffolds or hydrogels [87]. Scaffolds are solid, porous structures made from biocompatible materials such as natural or synthetic polymers. Native biomaterials offer not only higher biocompatibility but also higher batch-to-batch variability, while synthetic polymers are more easily reproducible but do not recapitulate the native properties of ECM [245]. On the other hand, hydrogels are crafted from biomimetic materials and categorized as naturally derived, synthetic, or hybrid hydrogels based on their origins and compositions [256]. Due to their hydrophilic properties, biocompatibility, and structural resemblance to the native cells’ microenvironment, hydrogels are a more reliable instrument for research with ECs.

The use of scaffold-based 3D models to study ECs behavior and molecular mechanisms is also more frequent in cancer research, namely to investigate angiogenesis associated with tumorigenesis, and still very scarce for ECD investigation in the context of CVDs [245, 247]. In one study, the effects of TNF-α stimulation were examined in HUVECs cultured in 2D TCPs and in a 3D hydrogel gelatin-based carotid artery model [257]. HUVECs grown in the 3D model exhibited not only a markedly elevated level of oxidative stress, expressed by an increased level of malondialdehyde and superoxide dismutase activity inhibition, but also an increased inflammatory response with more secretion of IL-1 and IL-6 in response to TNF-α when compared to ECs grown on 2D TCPs. Also, in the case of 3D cultures, Western blotting analysis demonstrated that angiotensin-converting enzyme and CD40 were upregulated, and that sirtuin 1 and sirtuin 6 were inhibited after TNF-α treatment. In addition, the effects of H_2_O_2_ were studied in HUVECs and, in particular, whether suppressing the expression of the polar protein partitioning defective protein 3 could affect the endothelial barrier function [258]. In this case, HUVECs in 3D culture were also more susceptible to damage by H_2_O_2_ than those cultured in 2D. Furthermore, analysis of different gene expression patterns showed that H_2_O_2_ altered cell–cell connections more markedly in 3D cultures by downregulating polarity-related signaling pathways in HUVECs through a reduced expression of claudin 1 gene and VE-cadherin, compromising the barrier function.

The scaffold-based 3D models mentioned above are considered static systems, as they replicate the structural support of the ECM but do not emulate the hemodynamic forces that ECs are exposed in vivo [225]. The models we mentioned previously to study shear stress are categorized as dynamic or flow-based cell culture systems but are still 2D models that lack the dimensionality provided by 3D models. However, there are dynamic scaffold-based 3D models used to study flow shear stress, such as polydimethylsiloxane (PDMS) only models, and others that combine the hydrogel cell culturing techniques with more complex microfluidic systems, namely PDMS-derived, PDMS-hydrogel hybrids, or pure hydrogel models [259].

PDMS-alone devices are usually stiff linear structures with microchannels where cells are seeded and grow lining the surface of the microchannel. These devices are permeable to gases and have an inlet-channel-outlet flow system; however, PDMS rigidity does not allow a full mimicry of the characteristics of native tissue environments, thus making no room for remodeling of ECs’ surroundings [216]. On the other side, PDMS-hydrogel hybrids and pure hydrogel models are much more successful in resembling the ECs’ microenvironment. In the case of PDMS-hydrogel hybrids, a middle section made of a hydrogel scaffold is lined between two outer sections that consist of PDMS channels lined with ECs [216]. The entire model is constructed to include microchannels that allow microcirculation of fluids regulated by external pump mechanisms, thus resulting in ECs’ migration from the PDMS surface onto the hydrogel giving rise to the formation of vessel sprouts. In the case of hydrogel-embedded microchannels, a PDMS chamber is created with two media orifices and an ECM orifice in the center containing a needle in the middle with a defined diameter from 15 to 300 μm [216]. Then, the hydrogel scaffold is allowed to be set at the ECM compartment around the needle that is removed once the polymerization of the scaffold is finished. This results in an open cylindrical microchannel integrated within the 3D scaffold where the ECs are seeded and grown until a completely endothelialized circular lumen is achieved [259]. These models have been widely used for studying angiogenesis and mechanisms behind shear stress-induced vascularization outside the context of ECD, but are also more focused on tumor angiogenesis [260, 261].

#### Organ-on-a-chip models

Over the past decade, the development of a novel technique known as organ-on-a-chip (OOAC) has gained great popularity [262, 263]. This technique typically comprises microfluidic devices combined with 3D microchip fabrication techniques, enabling the creation of continuously perfused chambers that mimic tissue and organ-level physiology [263]. These models have the ability not only of applying at a microscale what the models mentioned above employ in a macroscale but also, depending on their design, combine the best features of the 3D models: the dimensionality from hydrogels matrices resembling the ECM; the different hemodynamic forces that the manipulation of microfluids can simulate; and the possibility of including different chemical and biochemical gradients [264]. The PDMS-hydrogel hybrids and pure hydrogel models already described overlap with this definition of the OOAC technique. In reality, OOAC is more an application of these dynamic scaffold-based 3D models but with higher complexity. In the case of ECs, these models are sometimes also referred to as endothelium-on-a-chip, vessel-on-a-chip, or microvasculature-on-a-chip and sometimes require co-culturing ECs with vascular smooth muscle cells, fibroblasts, and other cells [265, 266].

Few works have applied these models to study ECD but not always in the context of CVDs. In a study that developed a model that included microfluidic channels in a type I collagen gel, HUVECs were seeded and cultured alone or with human brain vascular pericytes or human umbilical arterial smooth muscle cells embedded in the collagen scaffold [267]. The system included an inlet and an outlet system that allowed cells to seed and be perfused with culture medium or whole blood. The authors aimed to investigate interactions between ECs and perivascular cells, and interactions between blood components and endothelium in the context of angiogenesis and thrombosis. More relevant for targeting ECD was the study of blood–endothelium interactions, where HUVECs were divided into untreated (quiescent endothelium) and stimulated with phorbol-12-myristate-13-acetate (PMA) to mimic an inflammatory response before whole blood perfusion. In the untreated group, most platelets did not adhere and flowed past the endothelial surface. Only a minor fraction of platelets exhibited a reversible adhesion, later rolling along endothelial surfaces, while firm platelet adhesion was restricted to specific endothelial cell–cell junctions and areas of deformity within the endothelium. In the stimulated group, platelet aggregates rapidly formed on the endothelial surface, and continued to accumulate over time, while leukocytes adhered to the vessel walls and began migrating through the endothelium into the collagen matrix after 1 h of blood perfusion. To investigate thrombosis mechanisms, the collagen gel was perfused with fluorescein isothiocyanate (FITC)-conjugated antibodies against VWF. In unstimulated HUVECs, VWF was observed in the endothelial cytoplasm but not on the cells’ surfaces, whereas in stimulated vessels, long VWF fibers up to 2 mm in length covered the vessel surfaces, binding to platelets predominantly irreversibly. This 3D model was able to illustrate the inherent non-thrombotic characteristics of the vascular endothelium, and its shift toward a pro-thrombotic state during an inflammatory response.

A different study also developed a microfluidic device to mimic human microvascular networks using PDMS to culture HUVECs and analyzed the formation of an endothelial surface layer under a physiologically relevant level of fluid shear stress [268]. In this study, glycocalyx was observed in the entire lumen of the channels, making microchannels composed of stiff PDMS a suitable model for mechanistic studies.

Later, another study also aimed to create a model of microvasculature-on-a-chip based on a microfluidic device that included an agarose-gelatin gel where HUVECs, human dermal MVECs (HDMVECs), or human lung MVECs were cultured [266]. Researchers verified that all EC types maintained a functional, semi-permeable barrier in the hydrogel microchannels one-month post-seeding. They decided then to use this system to study the effects of pathologically altered red blood cells associated with sickle cell disease (SCD) and malaria, with or without associated inflammatory mediators, in the endothelial barrier function. Among other findings, they were able to confirm that: (i) overnight TNF-α perfusion increased permeability in HUVECs with upregulation of VCAM-1, ICAM-1, and E-selectin; (ii) 1 h perfusion of hemin (a hemolytic byproduct) at SCD relevant concentrations also increased permeability in HUVECs and HDMVECs; (iii) and perfusion of TNF-α followed by *Plasmodium falciparum*-infected red blood cells resulted in heightened occlusion of microchannels, with some vessels within the microvascular system being nearly completely obstructed. This endothelial permeability model can be easily utilized for investigating microvascular endothelial barrier function in CVDs.

In another investigation, a model was employed wherein gelatin methacryloyl hydrogel microchannels were either solely covered by HUVECs without fibroblasts (serving as the control), devoid of endothelial lining but containing encapsulated fibroblasts within the hydrogel, or with both endothelial lining along with encapsulated fibroblasts embedded within the matrix. [269]. The focus of this investigation was to study fibrosis. The microchannels were perfused with whole blood to form thrombi and then a microfluidic system was used to introduce a continuous flow of a thrombolytic agent. This study was able to confirm the model’s biomimetic nature in replicating the in vivo fibrosis, given the embedment of fibroblasts in the hydrogel, which migrated into the clot and led to collagen type I deposition. It serves as a good example of an OOAC with great potential for studying ECD and its relationship with vascular fibrosis.

OOAC models have been employed to study various vascular diseases such as atherosclerosis, thrombosis, pulmonary arterial hypertension, and others as extensively reviewed by Amid Shakeri et al*.* [270] and are a promising approach to model CVDs and to more accurately demystify the pathophysiology of ECD. However, one of the primary drawbacks of OOAC models is the limited number of cells that can be cultured within these small devices that often are too minute for traditional biochemical detection methods such as Western blotting, gel electrophoresis, and colorimetric detection [225]. These models are also highly complex to design. For example, in the case of ECs, each cell type, either cardiac or vascular, might have a preferential matrix for optimal culturing, and depending on the objective of the study, the microfluidic composition and dynamics must also be adjusted [270]. For these reasons, OOACs are difficult to validate, and sometimes, it is not possible to ensure the same experimental conditions between experiments. Nevertheless, they represent a great advance in investigation technology and their attempt to biomimicking cell and tissue environment is a crucial advantage compared to 2D cultures and a promising complement to animal model studies with great potential for further investigation of ECD in the context of CVDs.

## Conclusion

In vitro models of ECD allow the study of cellular and molecular mechanisms of disease and provide important research platforms for screening potential therapeutic agents. Even though other relevant options are available, such as animal models or ex vivo models, in vitro models offer higher experimental flexibility and reproducibility, making them a valuable tool for the understanding of pathophysiological mechanisms of several diseases, such as CVDs. The utilization of in vitro models becomes an even more appealing option if we take into account the growing ethical constraints on the use of animals and human samples in biomedical research. In sum, there are several in vitro models of ECD currently available, associated with the diversity of stimuli used to induce dysfunction. The use of a different cell type may require modification of the model, given the particular physiological characteristics of each endothelial cell type. It is important to note that, in CVDs, there is a complex interplay between different cells which must be considered. Thus, it would be important to develop more advanced in vitro models, where these interactions are accounted.

## Data Availability

No datasets were generated or analysed during the current study.
