# Correction to: Endothelial dysfunction in cardiovascular diseases: mechanisms and in vitro models

**DOI:** 10.1007/s11010-025-05390-0

**Published:** 2025-09-18

**Authors:** Ana Grego, Cristiana Fernandes, Ivo Fonseca, Marina Dias-Neto, Raquel Costa, Adelino Leite-Moreira, Sandra Marisa Oliveira, Fábio Trindade, Rita Nogueira-Ferreira

**Affiliations:** 1 RISE-Health, Department of Surgery and Physiology, Faculty of Medicine, University of Porto, Alameda Prof. Hernâni Monteiro, 4200-319 Porto, Portugal; 2 LAQV-REQUIMTE, Department of Chemistry, University of Aveiro, 3810-193 Aveiro, Portugal; 3 Department of Angiology and Vascular Surgery, Unidade Local de Saúde de São João, Alameda Prof. Hernâni Monteiro, 4200-319 Porto, Portugal; 4 Universidade Católica Portuguesa, CBQF-Centro de Biotecnologia e Química Fina-Laboratório Associado, Escola Superior de Biotecnologia, Rua Diogo Botelho 1327, 4169-005 Porto, Portugal; 5 Department of Cardiothoracic Surgery, Unidade Local de Saúde de São João, Alameda Prof. Hernâni Monteiro, 4200-319 Porto, Portugal

**Correction to: Molecular and Cellular Biochemistry (2025) 480:4671–4695** 10.1007/s11010-025-05289-w

In the original article the specific errors are as follows:

The sentences “Moreover, cardiac endothelium’s main energetic pathway is mitochondrial fatty acid oxidation [30]. In fact, it has been shown that genes that regulate fatty acid uptake, such as the Meox2/Tcf15, Fabp4, and Cd36, are highly upregulated in mice cardiac ECs [31]. In turn, ECs from the vascular endothelium (from both the macro and microcirculation) produce ATP mainly by glycolysis [32].” should be replaced by:

“Moreover, cardiac endothelium plays a crucial role in fatty acid uptake for cardiomyocytes, whose main energetic pathway is mitochondrial fatty acid oxidation [30]. In fact, it has been shown that genes that regulate fatty acid uptake, such as the Meox2/Tcf15, Fabp4, and Cd36, are highly upregulated in mice cardiac ECs [31]. ECs from the vascular endothelium (from both the macro and microcirculation) produce ATP mainly by glycolysis [32].”

Also, Fig. 1 should be replaced to remove the points “Predominant glycolysis” and “Predominant fatty acid oxidation”.

The Incorrect version of Fig. 1:

**Figure:**
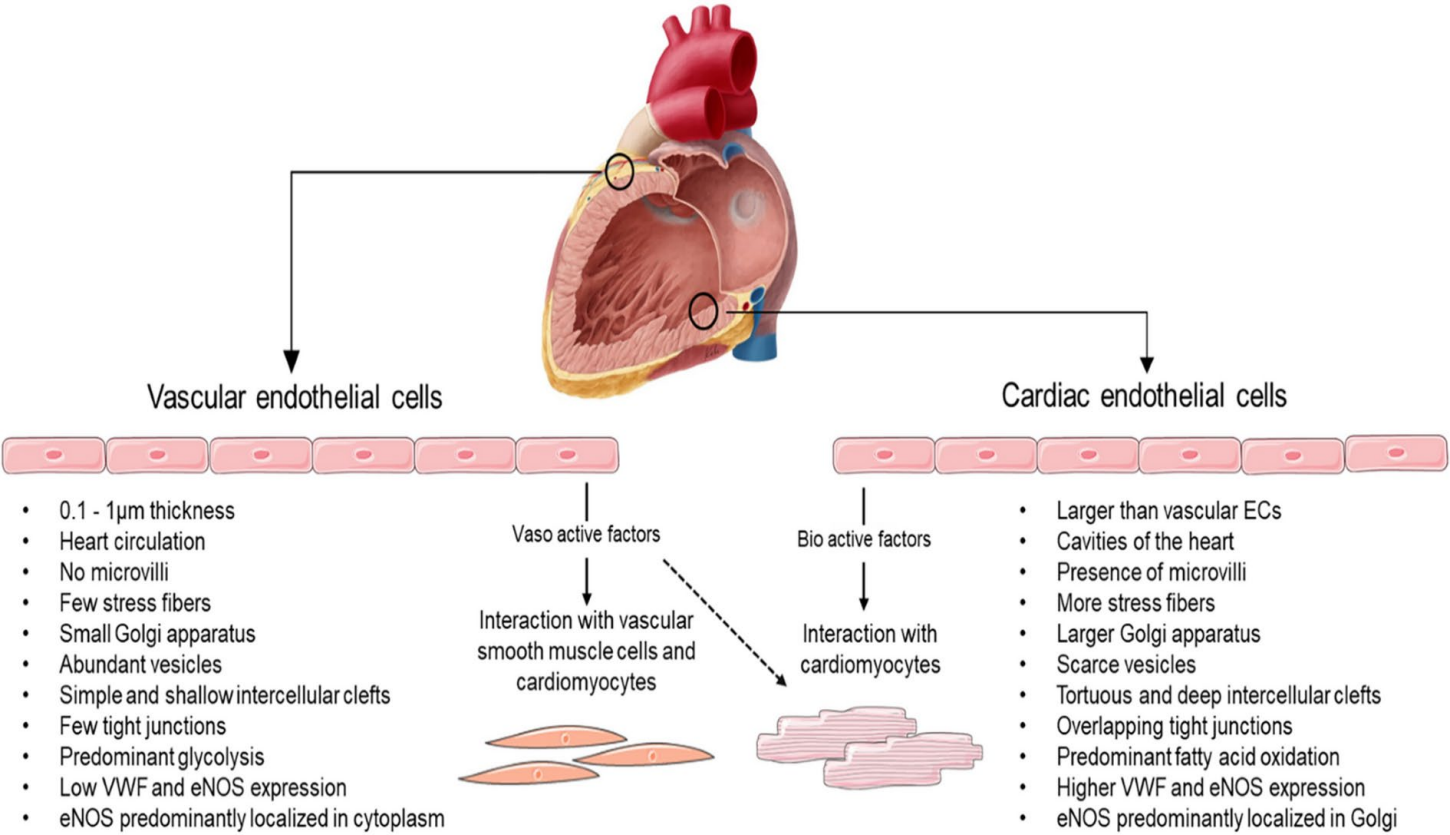

The Corrected version of Fig. 1:

**Fig. 1:**
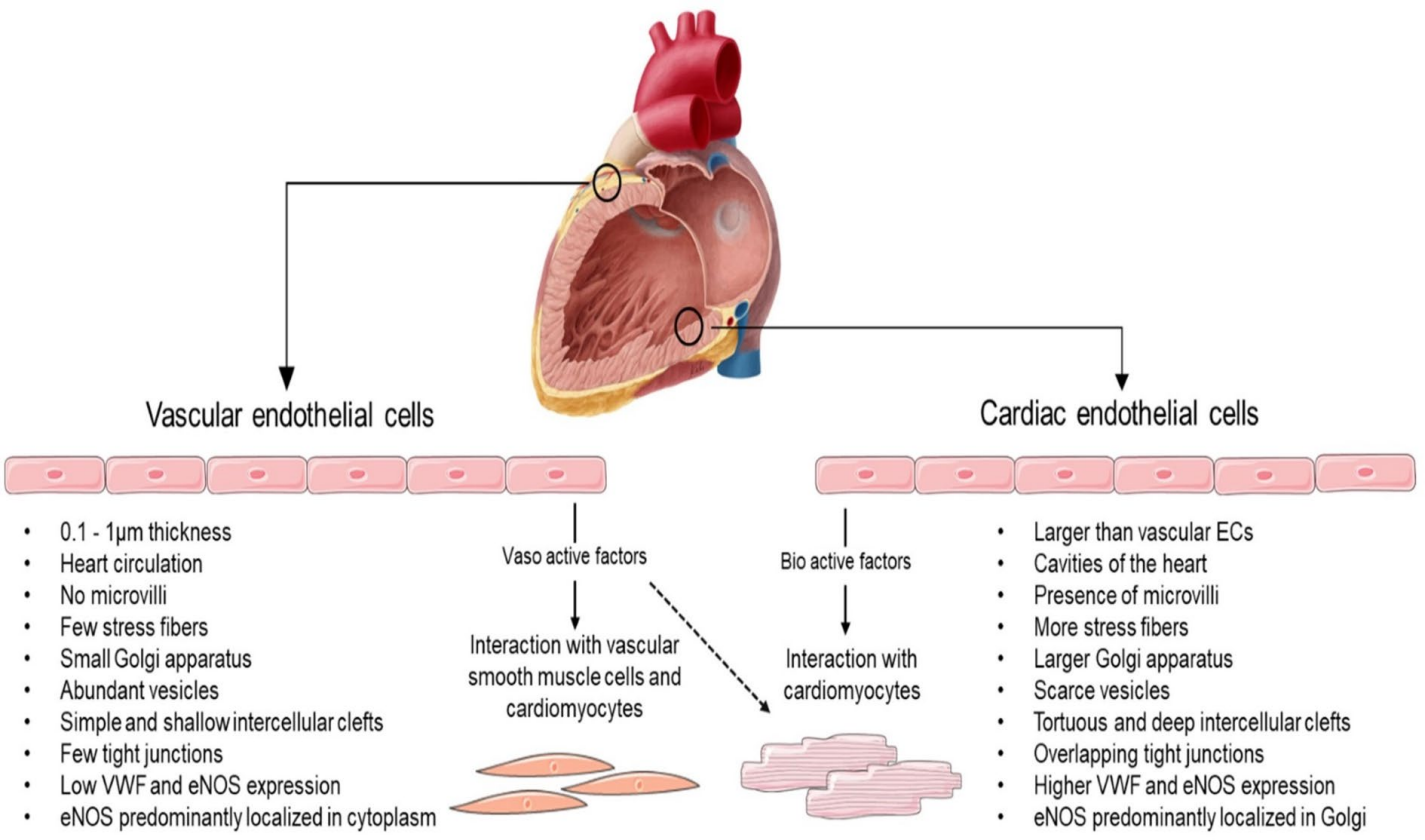
Characteristics of vascular and cardiac endothelial cells. Created with Smart Servier Medical Art (https://smart.servier.com/)

The original article has been corrected.

